# Ultrasound Guided Percutaneous Nephrolithotomy in Mesh-Repaired Incisional Hernia

**DOI:** 10.1155/2021/1537840

**Published:** 2021-11-28

**Authors:** Seyed Hassan Inanloo, Mohammad Reza Nikoobakht, Hamed Akhavizadegan, Mojgan Karbakhsh

**Affiliations:** ^1^Department of Urology, Sina Hospital, Tehran University of Medical Sciences, Tehran, Iran; ^2^Department of Community Medicine, Tehran University of Medical Sciences, Tehran, Iran

## Abstract

**Objectives:**

To describe our technique of percutaneous nephrolithotomy (PNL) in patients with mesh-repaired flank incisional hernia. Polypropylene mesh which is used for fascia strengthening in hernia repair elicits intense inflammatory reaction and the consequent fibrosis alters the characteristics of abdominal wall. Thus, prior history of flank hernia repair with mesh may result in percutaneous nephrolithotomy failure.

**Materials and Methods:**

Demographic data, renal stones characteristics, and any complication during surgery and follow-up of patients who were treated by PNL during 2011 to 2020 and had mesh in their flank region were collected.

**Results:**

Percutaneous nephrolithotomy was performed without any problem in 8 patients with guide of ultrasonography.

**Conclusion:**

Based on our experience, ultrasound-guided PNL is feasible and hypothetically superior to fluoroscopy in such circumstances.

## 1. Introduction

Introduction of polypropylene mesh was a great step in successful management of hernia and significantly decreased the likelihood of recurrence [1]. However, reoperation may be challenging in the mesh-covered regions due to intense inflammatory reaction and the consequent fibrosis alters the characteristics of abdominal wall [2]. Presence of scar tissue interferes with appropriate surgical access and the required tract dilatation during percutaneous nephrolithotomy (PNL) [3]. In these occasions, ultrasound can serve as a guide, visualizing the region, including the scar and fibrotic tissues. In this article, we report application of ultrasound for facilitating surgical access during PNL in a patient with history of mesh-based flank hernia repair.

## 2. Materials and Methods

During years 2011 to 2020, a total of 8 patients with history of flank incisional hernia which had been repaired by propylene mesh have been managed with ultrasound-guided percutaneous nephrolithotomy in our center. Age, gender, stones size and place, surgery adverse happenings, length of hospital stay, imaging after surgery, and follow-up visit 2 weeks and 2 months after surgery were recorded from their files anonymously.

**Figure 1:**
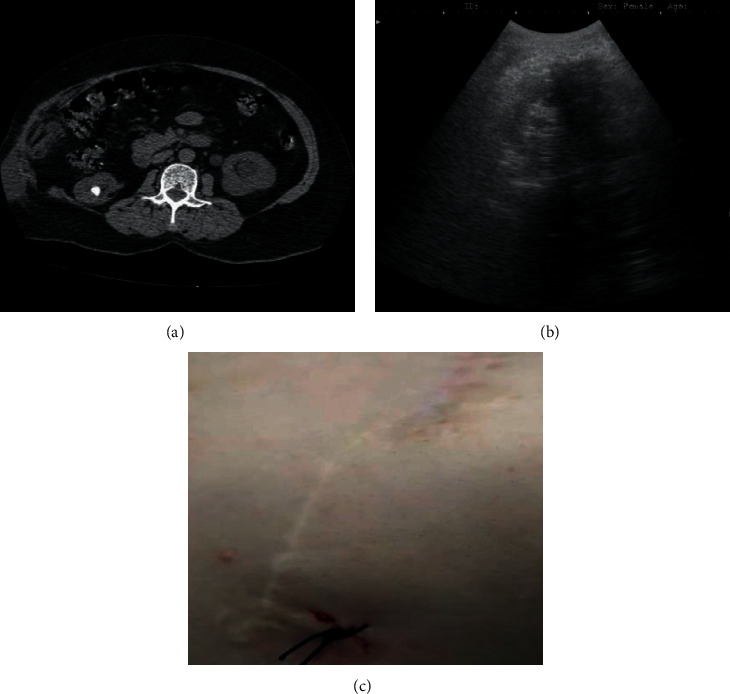
A 54-year-old woman with history of mesh-repaired right incisional flank hernia with right inferior calyx stone and left hydronephrosis; (a) CT scanning view, (b) ultrasound view of hypoechoic shadow of mesh, and (c) surgical hole of PNL and 2 incision lines in flank position for nephrolithotomy and hernia repair.

All of them had history of mesh-repaired flank incisional hernia due to previous nephrolithotomy. The main diagnostic modality before surgery was spiral CT scanning without contrast [4] (Figure 1(a)). Ultrasound was used to avoid puncture through the mesh in percutaneous nephrolithotomy in flank position [5, 6]. The mesh shadow on the right side of Figure 1(b) is seen as the hypoechoic area, while the kidney is clearly visible on the mesh-free side (i.e., the left side of the photo). Then, the mesh area was marked and the kidney was accessed successfully from lateral using the ultrasound guide. The puncture site in relation to the other two previous incisions (the old one in anterior and the recent one in posterior) is shown in Figure 1(c).

## 3. Results

3 males and 5 females, 48 to 65 year old were found. Three patients with a stone in the pelvis, 3 with inferior calyx stone, and the last 2 with stones in the inferior and middle calyx.

There was no complication during operation and 6 were stone free on follow-up imaging and 2 had residue smaller than 1 cm which were managed by extracorporeal shock wave lithotripsy. The patients were discharged as usual (2 and 3 days). There was no sign of infection of the mesh at early postoperative visit (2 weeks) and no sign of hernia recurrence at late postoperative visit (2 months).

## 4. Discussion

Percutaneous nephrolithotomy is a minimally invasive procedure which can be life-threatening in case of complications. Therefore, in order for the patient to enjoy the benefits of this minimally invasive method, it must be done with maximum observance of safety principles [7].

Following the use of mesh in incisional hernia repair, the abdominal wall becomes very stiff and fibrous and the intestines can be pulled to the location of the mesh in case of contact with the peritoneum [8]. Dilatation through the fibrotic area requires increased pressure on the dilators and Amplatz Sheath, which can damage the equipment or lead to loss of access and sudden/uncontrolled advancement of equipment into the kidney region through perforation [3].

For these reasons, approaching the area through access from the mesh-free zones is preferable. The areas affected by mesh fibrosis might not be easily discernible based on the external location of recent surgical scar, as the mesh sometimes extends to wider areas than the incision itself. As in our case, the mesh was radiolucent, using the C-arm, which was not useful either. Therefore, we decided to use ultrasound, which is an acceptable method of access in percutaneous nephrolithotomy [3].

Ultrasound not only has the ability to show if the bowels are pulled under the mesh but also provides a good view of the area covered by the mesh [9]. After finding a suitable, mesh-free site for the puncture, both C-arm and ultrasound can be used for the rest of the procedure. Due to the appropriate experience, we continued to work under the ultrasound guide [3].

Our study has its limitations. Inclusion criteria including history of open nephrolithotomy, herniation of the incision site, and the need for reoperation of the stone are the factors that reduce the number of patients. Another limitation of our study was the type of mesh used [10]. Because the patients had been operated on long ago in other centers, we did not know the exact type of mesh used except that the available mesh for incisional hernia repair at that time in our country was only nonabsorbable propylene mesh. However, due to the fibrosis and scar of the mesh site, which causes the strength of the surgical site and is visible in all types of meshes, the effect of the mesh type on the surgery will not be great and tract dilatation will be difficult in all types of meshes.

## 5. Conclusion

Based on our experience, ultrasound-guided PNL is feasible and hypothetically superior to fluoroscopy in case of percutaneous interventions with earlier mesh placement/involvement.

## Data Availability

We confirm that data and original pictures are available from the corresponding author upon request.
